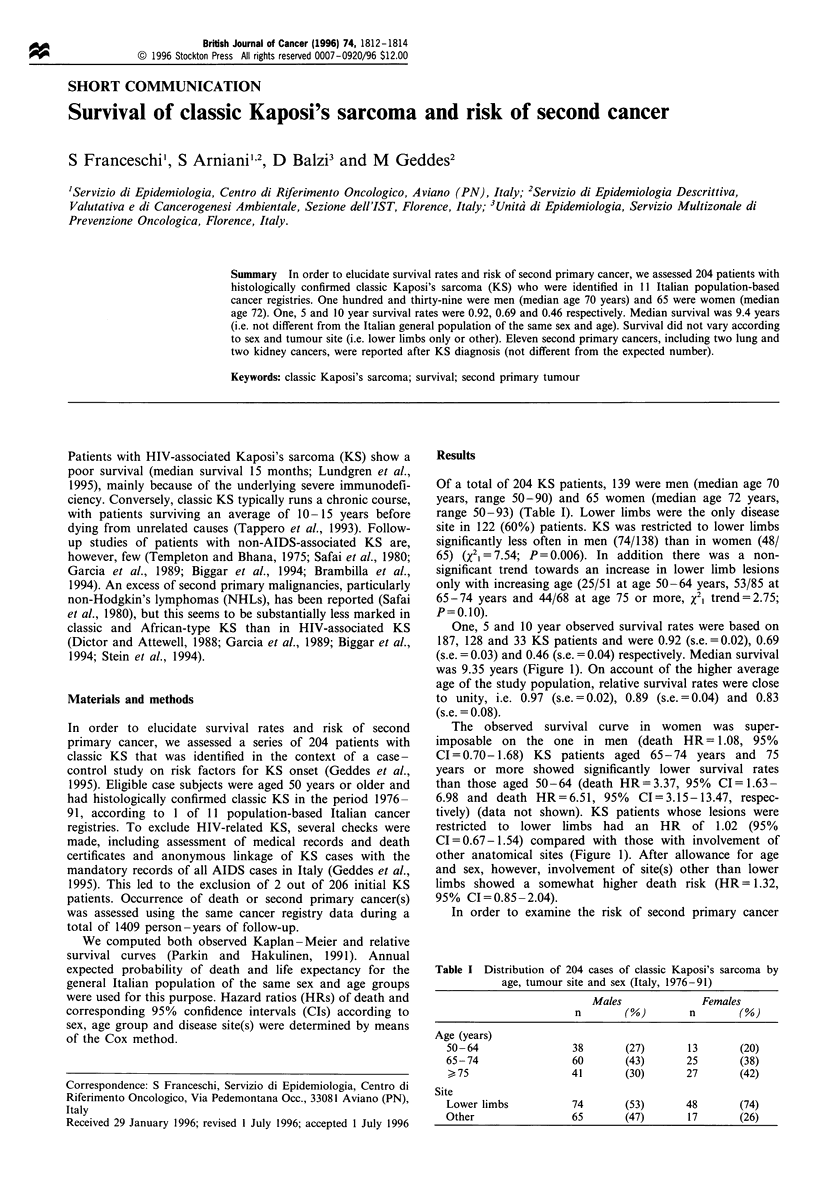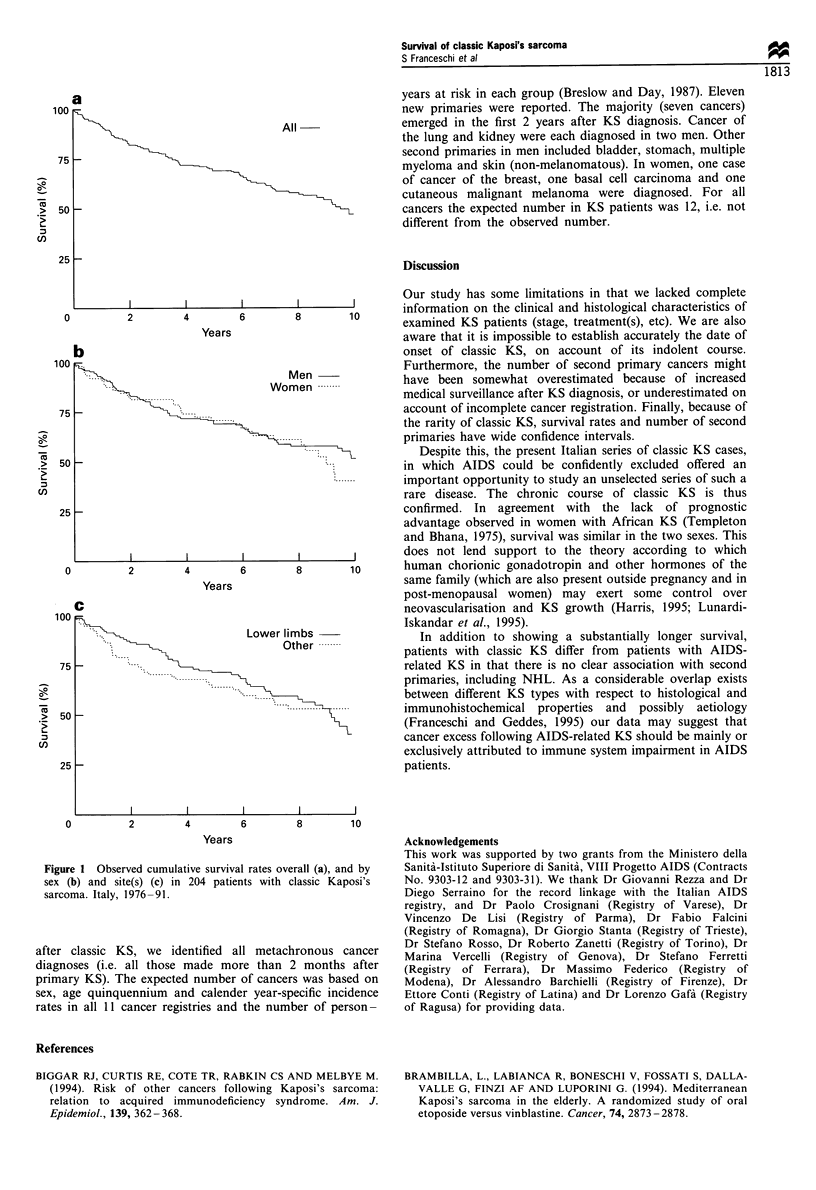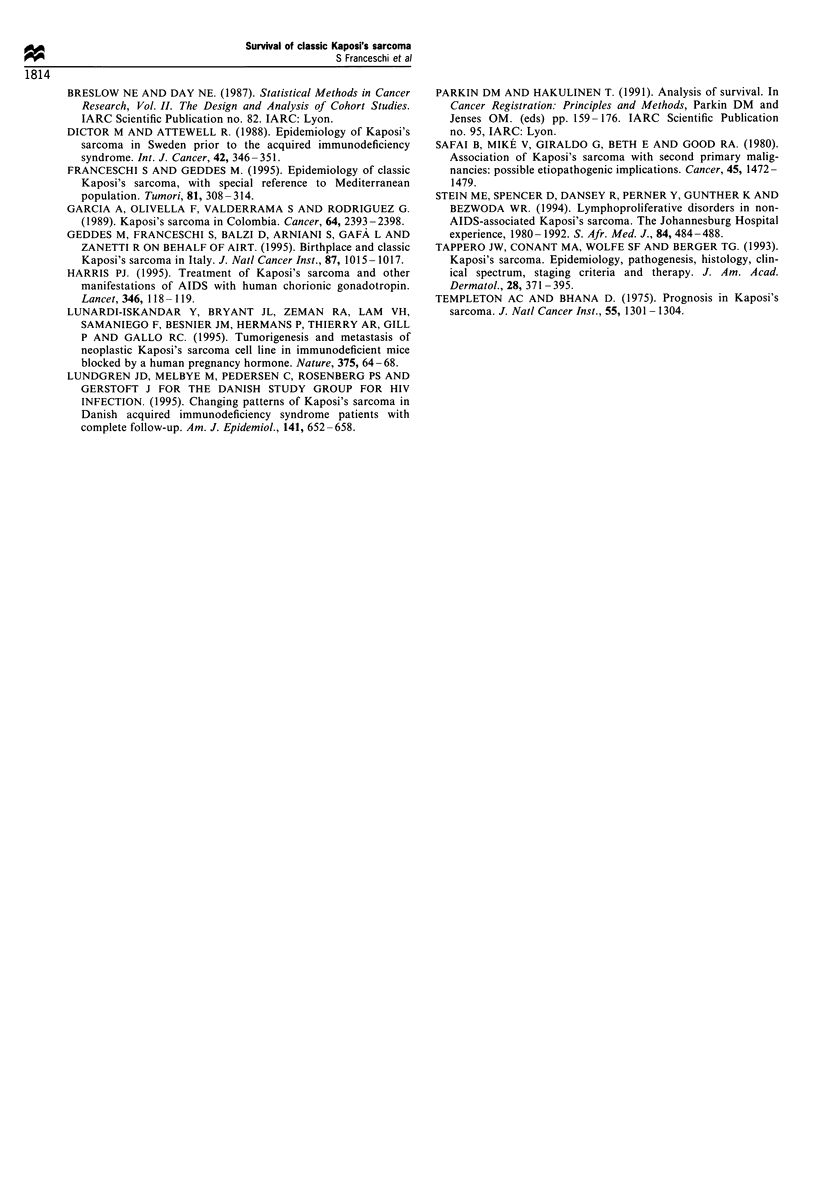# Survival of classic Kaposi's sarcoma and risk of second cancer.

**DOI:** 10.1038/bjc.1996.635

**Published:** 1996-12

**Authors:** S. Franceschi, S. Arniani, D. Balzi, M. Geddes

**Affiliations:** Servizio di Epidemiologia, Centro di Riferimento Oncologico, Aviano (PN), Italy.

## Abstract

In order to elucidate survival rates and risk of second primary cancer, we assessed 204 patients with histologically confirmed classic Kaposi's sarcoma (KS) who were identified in 11 Italian population-based cancer registries. One hundred and thirty-nine were men (median age 70 years) and 65 were women (median age 72). One, 5 and 10 year survival rates were 0.92, 0.69 and 0.46 respectively. Median survival was 9.4 years (i.e. not different from the Italian general population of the same sex and age). Survival did not vary according to sex and tumour site (i.e. lower limbs only or other). Eleven second primary cancers, including two lung and two kidney cancers, were reported after KS diagnosis (not different from the expected number).


British Journal of Cancer (1996) 74, 1812-1814
© 1996 Stockton Press All rights reserved 0007-0920/96 $12.00

SHORT COMMUNICATION

### Survival of classic Kaposi's sarcoma and risk of second cancer

S Franceschi[1], S Arniani[1,2], D Balzi[3] and M Geddes[2]

[1]*Servizio di Epidemiologia, Centro di Riferimento Oncologico, Aviano (PN), Italy;* [2]*Servizio di Epidemiologia Descrittiva, Valutativa e di Cancerogenesi Ambientale, Sezione dell'IST, Florence, Italy;* [3]*Unità di Epidemiologia, Servizio Multizonale di Prevenzione Oncologica, Florence, Italy.*

**Summary** In order to elucidate survival rates and risk of second primary cancer, we assessed 204 patients with histologically confirmed classic Kaposi's sarcoma (KS) who were identified in 11 Italian population-based cancer registries. One hundred and thirty-nine were men (median age 70 years) and 65 were women (median age 72). One, 5 and 10 year survival rates were 0.92, 0.69 and 0.46 respectively. Median survival was 9.4 years (i.e. not different from the Italian general population of the same sex and age). Survival did not vary according to sex and tumour site (i.e. lower limbs only or other). Eleven second primary cancers, including two lung and two kidney cancers, were reported after KS diagnosis (not different from the expected number).

**Keywords:** classic Kaposi's sarcoma; survival; second primary tumour

Patients with HIV-associated Kaposi's sarcoma (KS) show a poor survival (median survival 15 months; Lundgren *et al.*, 1995), mainly because of the underlying severe immunodeficiency. Conversely, classic KS typically runs a chronic course, with patients surviving an average of 10-15 years before dying from unrelated causes (Tappero *et al.*, 1993). Follow-up studies of patients with non-AIDS-associated KS are, however, few (Templeton and Bhana, 1975; Safai *et al.*, 1980; Garcia *et al.*, 1989; Biggar *et al.*, 1994; Brambilla *et al.*, 1994). An excess of second primary malignancies, particularly non-Hodgkin's lymphomas (NHLs), has been reported (Safai *et al.*, 1980), but this seems to be substantially less marked in classic and African-type KS than in HIV-associated KS (Dictor and Attewell, 1988; Garcia *et al.*, 1989; Biggar *et al.*, 1994; Stein *et al.*, 1994).

### Materials and methods

In order to elucidate survival rates and risk of second primary cancer, we assessed a series of 204 patients with classic KS that was identified in the context of a case-control study on risk factors for KS onset (Geddes *et al.*, 1995). Eligible case subjects were aged 50 years or older and had histologically confirmed classic KS in the period 1976-91, according to 1 of 11 population-based Italian cancer registries. To exclude HIV-related KS, several checks were made, including assessment of medical records and death certificates and anonymous linkage of KS cases with the mandatory records of all AIDS cases in Italy (Geddes *et al.*, 1995). This led to the exclusion of 2 out of 206 initial KS patients. Occurrence of death or second primary cancer(s) was assessed using the same cancer registry data during a total of 1409 person-years of follow-up.

We computed both observed Kaplan-Meier and relative survival curves (Parkin and Hakulinen, 1991). Annual expected probability of death and life expectancy for the general Italian population of the same sex and age groups were used for this purpose. Hazard ratios (HRs) of death and corresponding 95% confidence intervals (CIs) according to sex, age group and disease site(s) were determined by means of the Cox method.

Correspondence: S Franceschi, Servizio di Epidemiologia, Centro di Riferimento Oncologico, Via Pedemontana Occ., 33081 Aviano (PN), Italy
Received 29 January 1996; revised 1 July 1996; accepted 1 July 1996

### Results

Of a total of 204 KS patients, 139 were men (median age 70 years, range 50-90) and 65 women (median age 72 years, range 50-93) (Table I). Lower limbs were the only disease site in 122 (60%) patients. KS was restricted to lower limbs significantly less often in men (74/138) than in women (48/65) ($\chi^2_1 = 7.54$; $P = 0.006$). In addition there was a non-significant trend towards an increase in lower limb lesions only with increasing age (25/51 at age 50-64 years, 53/85 at 65-74 years and 44/68 at age 75 or more, $\chi^2_1$ trend = 2.75; $P = 0.10$).

One, 5 and 10 year observed survival rates were based on 187, 128 and 33 KS patients and were 0.92 (s.e. = 0.02), 0.69 (s.e. = 0.03) and 0.46 (s.e. = 0.04) respectively. Median survival was 9.35 years (Figure 1). On account of the higher average age of the study population, relative survival rates were close to unity, i.e. 0.97 (s.e. = 0.02), 0.89 (s.e. = 0.04) and 0.83 (s.e. = 0.08).

The observed survival curve in women was superimposable on the one in men (death HR = 1.08, 95% CI = 0.70-1.68) KS patients aged 65-74 years and 75 years or more showed significantly lower survival rates than those aged 50-64 (death HR = 3.37, 95% CI = 1.63-6.98 and death HR = 6.51, 95% CI = 3.15-13.47, respectively) (data not shown). KS patients whose lesions were restricted to lower limbs had an HR of 1.02 (95% CI = 0.67-1.54) compared with those with involvement of other anatomical sites (Figure 1). After allowance for age and sex, however, involvement of site(s) other than lower limbs showed a somewhat higher death risk (HR = 1.32, 95% CI = 0.85-2.04).

In order to examine the risk of second primary cancer

**Table I** Distribution of 204 cases of classic Kaposi's sarcoma by age, tumour site and sex (Italy, 1976-91)

| | *Males* n | *Males* (%) | *Females* n | *Females* (%) |
|---|---|---|---|---|
| Age (years) | | | | |
| 50-64 | 38 | (27) | 13 | (20) |
| 65-74 | 60 | (43) | 25 | (38) |
| ≥75 | 41 | (30) | 27 | (42) |
| Site | | | | |
| Lower limbs | 74 | (53) | 48 | (74) |
| Other | 65 | (47) | 17 | (26) |

**Figure 1** Observed cumulative survival rates overall (**a**), and by sex (**b**) and site(s) (**c**) in 204 patients with classic Kaposi's sarcoma. Italy, 1976–91.

after classic KS, we identified all metachronous cancer diagnoses (i.e. all those made more than 2 months after primary KS). The expected number of cancers was based on sex, age quinquennium and calender year-specific incidence rates in all 11 cancer registries and the number of person–years at risk in each group (Breslow and Day, 1987). Eleven new primaries were reported. The majority (seven cancers) emerged in the first 2 years after KS diagnosis. Cancer of the lung and kidney were each diagnosed in two men. Other second primaries in men included bladder, stomach, multiple myeloma and skin (non-melanomatous). In women, one case of cancer of the breast, one basal cell carcinoma and one cutaneous malignant melanoma were diagnosed. For all cancers the expected number in KS patients was 12, i.e. not different from the observed number.

### Discussion

Our study has some limitations in that we lacked complete information on the clinical and histological characteristics of examined KS patients (stage, treatment(s), etc). We are also aware that it is impossible to establish accurately the date of onset of classic KS, on account of its indolent course. Furthermore, the number of second primary cancers might have been somewhat overestimated because of increased medical surveillance after KS diagnosis, or underestimated on account of incomplete cancer registration. Finally, because of the rarity of classic KS, survival rates and number of second primaries have wide confidence intervals.

Despite this, the present Italian series of classic KS cases, in which AIDS could be confidently excluded offered an important opportunity to study an unselected series of such a rare disease. The chronic course of classic KS is thus confirmed. In agreement with the lack of prognostic advantage observed in women with African KS (Templeton and Bhana, 1975), survival was similar in the two sexes. This does not lend support to the theory according to which human chorionic gonadotropin and other hormones of the same family (which are also present outside pregnancy and in post-menopausal women) may exert some control over neovascularisation and KS growth (Harris, 1995; Lunardi-Iskandar *et al.*, 1995).

In addition to showing a substantially longer survival, patients with classic KS differ from patients with AIDS-related KS in that there is no clear association with second primaries, including NHL. As a considerable overlap exists between different KS types with respect to histological and immunohistochemical properties and possibly aetiology (Franceschi and Geddes, 1995) our data may suggest that cancer excess following AIDS-related KS should be mainly or exclusively attributed to immune system impairment in AIDS patients.

**Acknowledgements**
This work was supported by two grants from the Ministero della Sanità-Istituto Superiore di Sanità, VIII Progetto AIDS (Contracts No. 9303-12 and 9303-31). We thank Dr Giovanni Rezza and Dr Diego Serraino for the record linkage with the Italian AIDS registry, and Dr Paolo Crosignani (Registry of Varese), Dr Vincenzo De Lisi (Registry of Parma), Dr Fabio Falcini (Registry of Romagna), Dr Giorgio Stanta (Registry of Trieste), Dr Stefano Rosso, Dr Roberto Zanetti (Registry of Torino), Dr Marina Vercelli (Registry of Genova), Dr Stefano Ferretti (Registry of Ferrara), Dr Massimo Federico (Registry of Modena), Dr Alessandro Barchielli (Registry of Firenze), Dr Ettore Conti (Registry of Latina) and Dr Lorenzo Gafà (Registry of Ragusa) for providing data.

### References

BIGGAR RJ, CURTIS RE, COTE TR, RABKIN CS AND MELBYE M. (1994). Risk of other cancers following Kaposi's sarcoma: relation to acquired immunodeficiency syndrome. *Am. J. Epidemiol.*, **139**, 362–368.

BRAMBILLA, L., LABIANCA R, BONESCHI V, FOSSATI S, DALLA-VALLE G, FINZI AF AND LUPORINI G. (1994). Mediterranean Kaposi's sarcoma in the elderly. A randomized study of oral etoposide versus vinblastine. *Cancer*, **74**, 2873–2878.

BRESLOW NE AND DAY NE. (1987). *Statistical Methods in Cancer Research, Vol. II. The Design and Analysis of Cohort Studies.* IARC Scientific Publication no. 82. IARC: Lyon.

DICTOR M AND ATTEWELL R. (1988). Epidemiology of Kaposi's sarcoma in Sweden prior to the acquired immunodeficiency syndrome. *Int. J. Cancer*, **42**, 346–351.

FRANCESCHI S AND GEDDES M. (1995). Epidemiology of classic Kaposi's sarcoma, with special reference to Mediterranean population. *Tumori*, **81**, 308–314.

GARCIA A, OLIVELLA F, VALDERRAMA S AND RODRIGUEZ G. (1989). Kaposi's sarcoma in Colombia. *Cancer*, **64**, 2393–2398.

GEDDES M, FRANCESCHI S, BALZI D, ARNIANI S, GAFÀ L AND ZANETTI R ON BEHALF OF AIRT. (1995). Birthplace and classic Kaposi's sarcoma in Italy. *J. Natl Cancer Inst.*, **87**, 1015–1017.

HARRIS PJ. (1995). Treatment of Kaposi's sarcoma and other manifestations of AIDS with human chorionic gonadotropin. *Lancet*, **346**, 118–119.

LUNARDI-ISKANDAR Y, BRYANT JL, ZEMAN RA, LAM VH, SAMANIEGO F, BESNIER JM, HERMANS P, THIERRY AR, GILL P AND GALLO RC. (1995). Tumorigenesis and metastasis of neoplastic Kaposi's sarcoma cell line in immunodeficient mice blocked by a human pregnancy hormone. *Nature*, **375**, 64–68.

LUNDGREN JD, MELBYE M, PEDERSEN C, ROSENBERG PS AND GERSTOFT J FOR THE DANISH STUDY GROUP FOR HIV INFECTION. (1995). Changing patterns of Kaposi's sarcoma in Danish acquired immunodeficiency syndrome patients with complete follow-up. *Am. J. Epidemiol.*, **141**, 652–658.

PARKIN DM AND HAKULINEN T. (1991). Analysis of survival. In *Cancer Registration: Principles and Methods*, Parkin DM and Jenses OM. (eds) pp. 159–176. IARC Scientific Publication no. 95, IARC: Lyon.

SAFAI B, MIKÉ V, GIRALDO G, BETH E AND GOOD RA. (1980). Association of Kaposi's sarcoma with second primary malignancies: possible etiopathogenic implications. *Cancer*, **45**, 1472–1479.

STEIN ME, SPENCER D, DANSEY R, PERNER Y, GUNTHER K AND BEZWODA WR. (1994). Lymphoproliferative disorders in non-AIDS-associated Kaposi's sarcoma. The Johannesburg Hospital experience, 1980–1992. *S. Afr. Med. J.*, **84**, 484–488.

TAPPERO JW, CONANT MA, WOLFE SF AND BERGER TG. (1993). Kaposi's sarcoma. Epidemiology, pathogenesis, histology, clinical spectrum, staging criteria and therapy. *J. Am. Acad. Dermatol.*, **28**, 371–395.

TEMPLETON AC AND BHANA D. (1975). Prognosis in Kaposi's sarcoma. *J. Natl Cancer Inst.*, **55**, 1301–1304.